# Carboxyl-terminal truncated HBx contributes to invasion and metastasis via deregulating metastasis suppressors in hepatocellular carcinoma

**DOI:** 10.18632/oncotarget.10399

**Published:** 2016-07-04

**Authors:** Weihua Li, Man Li, Dongjiang Liao, Xinpeng Lu, Xia Gu, Qianqian Zhang, Zhixiang Zhang, Hui Li

**Affiliations:** ^1^ Department of Gastroenterology, Zhujiang Hospital of Nanfang Medical University, Guangzhou 510280, China; ^2^ Department of Infectious Disease and Hepatology, Hepatitis Research Room, The First Affiliated Hospital of Guangzhou Medical University, Guangzhou 510120, China; ^3^ Pathology Research Room, State Key Laboratory of Respiratory Disease, Guangzhou Institute of Respiratory Disease, Guangzhou 510120, China; ^4^ Department of Pathology, The First Affiliated Hospital of Guangzhou Medical University, Guangzhou 510120, China

**Keywords:** HBV, C-terminal truncated HBx, metastasis-suppressors, transcriptional regulation, hepatocellular carcinoma

## Abstract

Hepatitis B virus (HBV) X protein (HBx), a trans-regulator, is frequently expressed in truncated form without carboxyl-terminus in hepatocellular carcinoma (HCC), but its functional mechanisms are not fully defined. In this report, we investigated frequency of this natural HBx mutant in HCCs and its functional significance. In 102 HBV-infected patients with HCC, C-terminal truncation of HBx, in contrast to full-length HBx, were more prevalent in tumors (70.6%) rather than adjacent non-tumorous tissues (29.4%) (*p =* 0.0032). Furthermore, two naturally-occurring HBx variants (HBxΔ31), which have 31 amino acids (aa) deleted (codons 123-125/124-126) at C-terminus were identified in tumors and found that the presence of HBxΔ31 significantly correlated with intrahepatic metastasis. We also show that over-expression of HBxΔ31 enhanced hepatoma cell invasion *in vitro* and metastasis *in vivo* compared to full-length HBx. Interestingly, HBxΔ31 exerts this function via down-regulating Maspin, RhoGDIα and CAPZB, a set of putative metastasis-suppressors in HCC, in part, by enhancing the binding of transcriptional repressor, myc-associated zinc finger protein (MAZ) to the promoters through physical association with MAZ. Notably, these HBxΔ31-repressed proteins were also significantly lower expression in a subset of HCC tissues with C-terminal HBx truncation than the adjacent non-tumorous tissues, highlighting the clinical significance of this novel HBxΔ31-driven metastatic molecular cascade. Our data suggest that C-terminal truncation of HBx, particularly breakpoints at 124aa, plays a role in enhancing hepatoma cell invasion and metastasis by deregulating a set of metastasis-suppressors partially through MAZ, thus uncovering a novel mechanism for the progression of HBV-associated hepatocarcinogenesis.

## INTRODUCTION

Hepatocellular carcinoma (HCC) is a complex and heterogeneous disease involved in multiple-stages and multiple-factors. The high rate of metastasis or post-operation recurrence and the low response rate to chemotherapy are responsible for poor prognosis of HCC patients [1]. Despite many risk factors account for the development of HCC, the growing evidence indicated that chronic and persistent infection with hepatitis B virus (HBV) is a major leading cause, particularly in China, where more than 80% of HCC cases has a history of HBV infection [1]. One of the open-reading frames encoded by the HBV genome, a multifunctional regulatory X protein (HBx), is believed to play vital roles in HBV-induced tumorigenesis [2–4]. As a trans-regulatory factor, HBx has promiscuous functions both *in vitro* and *in vivo*, including transactivation of cellular and viral promoters, regulation of signaling pathways, promotion of cell cycle progression, alteration of apoptosis, and damage of cellular DNA repair [2]. Therefore, HBx is likely to be involved in the several different steps of the development and progression of HCC [3]. In addition, immortalized hepatocytes stably transfected with the HBV genome form metastatic tumors upon inoculation in nude mice [4]. HBx mRNA selectively accumulates in HCCs from hepatitis B surface antigen-negative patients [5], suggesting a critical role for HBx in late events of the carcinoma development.

The random integration of HBV led to the truncation of viral proteins, particularly at the C-terminal ends of HBx [6–9]. These natural C-terminal truncated HBx (Ct-HBx), frequently expressed in HBV-associated HCCs, have been shown to abrogate the growth-suppressive effects induced by full-length HBx, increase the proliferation of neoplastic cell, and promote the transforming ability of hepatocytes [9–11]. Thus, Ct-HBx may play a important role in facilitating hepatocarcinogenesis via modifying biological functions of full-length HBx. Our and others groups have described that the full-length HBx participates in diverse process of HCC progression through inducing alternation of cellular morphology, disruption of adherens junction and epithelial-mesenchymal transition [10–14], but whether Ct-HBx play a role in HCC metastasis and the underlying mechanisms is little known. Although recent reports showed that Ct-HBx mutants can promote cell invasion and metastasis of HCC by up-regulating matrix metalloproteinase10 or down-regulating Wnt-5α [15, 16], in contrast to the full-length HBx, our understanding to functional contribution of C-terminal truncation of HBx during the progression of hepatocarcinigenesis is limited so far.

In the present study, we found that C-terminal truncation of HBx, particularly with the breakpoints at 124aa (HBxΔ31), is frequently expressed in HBV-related HCC and significantly associated with intrahepatic metastasis. We also show that ectopic expression of HBxΔ31 enhanced hepatoma cell invasion *in vitro* and metastasis *in vivo* by deregulating a set of putative metastasis-suppressors in HCC, in part through enhancing MAZ to its consensus sequence in the promoters. Notably, these HBxΔ31-repressed proteins were also lower expression in HCC tumors with Ct-HBx than the adjacent non-tumorous liver tissues and indicated poor prognosis in HCC patients. These results provide new insights into the molecular mechanisms of metastasis-suppressors in response to C-terminal truncation of HBx, revealing the importance of this novel HBxΔ31-driven metastatic molecular cascade during the metastasis of HBV-associated HCC.

## RESULTS

### Over-expression of Ct-HBx in HCC tissues is associated with intrahepatic metastasis

**Figure 1:**
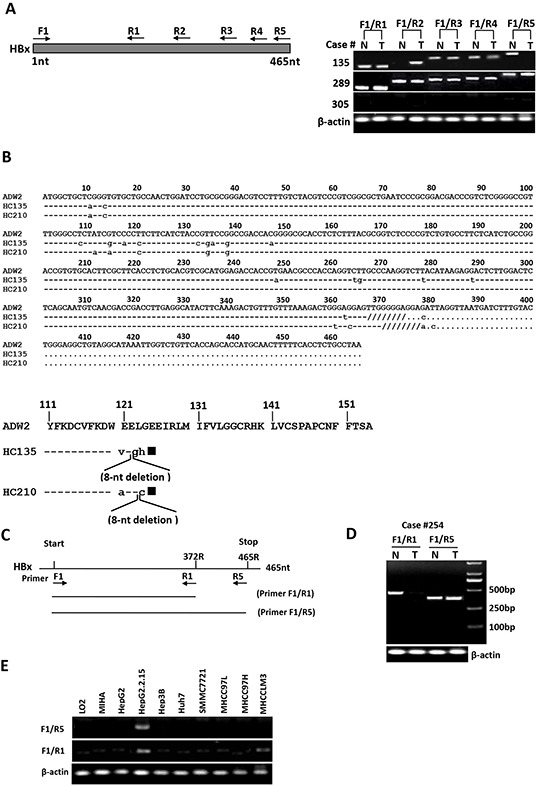
Detection of full-length and C-terminal truncated forms of HBx DNA in human HCC samples and mRNA in human hepatoma cell lines **A.** Representative results showed the presence of C-terminal truncated and full-length HBx DNA in HCC tumors (T) and their corresponding non-tumorous liver tissues (N). Case 135 had C-terminal truncated HBx DNA in tumor and full-length HBx in non-tumorous tissue. Case 289 showed the presence of full-length and C-terminal truncated HBx in both tumor and non-tumorous tissue, whereas case 305 was from an HBsAg-negative patient and showed no HBx DNA in both tumor and non-tumorous liver tissue. **B.** Sequencing analysis of PCR products showed the presence of two specific substantial deletions within HBx DNA from HCC tissues. On the upper panel: sequence alignment of full-length HBx DNA from a control sequence of HBV adw2 subtypes (GenBank accession number X02763) and case 135 with the deletion at nt 367-374 (codons 123-125), and case 210 with the deletion at nt 370-377 (codons 124-126). The 8-nucleotide deletions are shown as “////////”. Lower letter represents the substantial nucleotide. On the lower panel: alignment of deduced amino acids (aa) at C-terminal end of HBx DNA from a control sequence of HBV adw2 (154aa) and HCC tissues of two patients (cases 135 and 210). Case 135: deletion at nt 367-374, frame shift and new stop codon formation, resulting in loss of 34aa and formation of 3 new amino acids. Case 210: deletion at nt 370-377, frame shift and new stop codon formation, resulting in loss of 33aa and formation of 2 new amino acids. The expected HBx protein of cases 135 and 210 each would be 31aa shorter than full-length HBx protein, denoted as HBxΔ31. Solid square represents new stop codon formed due to deletion and frame shift in HBx DNA. Lower letter represents the new amino acids. **C.** Binding locations of two PCR primer pairs targeting the full-length (F1-R5) and 3′-deleted (F1-R1) HBx gene. **D.** One representative example of HCC cases with HBx truncation in tumors, wherein the short HBx fragment were amplified from both tumor (T) and non-tumorous (N) tissues, but full-length HBx fragment was amplified only from adjacent non-tumorous liver tissues. β-actin was used as an internal control. **E.** RT-PCR analysis in HCC cell lines showed the presence of HBxΔ31 transcripts only in MHCC-LM3 cells, but not in other HCC cell lines or the immortalized normal liver cell lines.

To investigate whether the presence of natural Ct-HBx mutant is correlated with metastasis in HCC, we first examined HBx integrity (full-length or truncation) in the 102 HBV-positive HCC patients by PCR analysis using 5 pairs of PCR primers franking the different lengths of HBx sequence (Figure 1A). In all 102 HBV-positive patients, full-length HBx was frequently found in the non-tumorous tissues (92 of 102, 90.2%) but was rarely detected in HCC tissues (30 of 102, 29.4%; *p* < 0.0001). In contrast, natural C-terminal truncation of HBx was more prevalent in HCC tumors (72 of 102, 70.6%) than in the adjacent non-tumorous liver tissues (46 of 102, 45%; *p =* 0.0032), indicating the presence of Ct-HBx is a frequent event in HCC (Figure 1A). Moreover, the HBx DNA breakpoints between 120aa to 130aa was the major form of truncation, being found in 45.8% (33/72) of the cases (Supplementary Figure S1). Interestingly, we identified two substantial-deletions in HBx gene from HCC tissues. The first deletion was at nucleotides (nt) 367-374 (codons 123-125) of HBx DNA. One nucleotide substitution produced a frame shift and a new stop codon (TAG) formation at the downstream (nt 381-383), leading to loss of 34aa and formation of 3 new amino acids at C-terminus. The second deletion was at nt 370-377 (codons 124-126) of HBx DNA. Two nucleotides substitution gave rise to a frame shift and a new stop codon (TAG) formation at the downstream (nt 381-383), resulting in loss of 33aa and formation of 2 new amino acids at C-terminus (Figure 1B). These two mutations generated HBx protein with 31aa shorter than full-length HBx, namely HBxΔ31.

To further explore the functional significance of HBxΔ31 in development and progression of HCC, we analyzed expression of HBxΔ31 from DNA samples of the same 102 paired clinical HCC specimen using two pairs of PCR primers flanking different sequences of full-length HBx and HBxΔ31 (Figure 1C and 1D). As shown in Figure 1D, HBxΔ31 expression was frequently present in HCC tumors (78 of 102, 76.5%) compared to in the adjacent non-tumorous liver tissues (35 of 102, 34.3%; *p* < 0.001) (Figure 1D). Next, we analyzed the correlation between HBxΔ31 expression and clinicopathological features of HBV-related HCC. We found that HBxΔ31 expression was significantly associated with intrahepatic metastasis of HCC (Table 1), suggesting that the presence of HBxΔ31 in HCCs was involved in the progression of hepatocarcinogenesis.

**Table 1:** Correlation between HBxΔ31, Maspin, RhoGDIα and CAPZB expression and clinicopathological features in human HCCs

Characteristics	Tumor HBxΔ31 expression		P value	Tumor Maspin expression		P value	Tumor RhoGDIα expression		P value	Tumor CAPZB expression		P value
	Negative (34)	Positive (68)		Negative (52)	Positive (50)		Negative (55)	Positive (47)		Negative (62)	Positive (40)	
Age (yr)	50.5 (7.9)	51.4 (8.1)		50.9 (8.1)	50.1 (8.5)		51.8 (8.2)	50.5 (8.9)		50.9 (8.4)	52.1 (8.1)	
Gender												
Male	25	51	0.421	45	39	0.642	39	37	0.198	40	36	0.559
Female	10	16		12	6		16	10		18	8	
Serum AFP												
<20ng/mL	15	27	0.887	37	26	0.121	33	30	0.195	35	26	0.87
≥20ng/mL	21	39		25	14		20	19		20	21	
Cirrhosis												
Absent	15	28	0.061	25	14	0.78	22	20	0.188	24	21	0.112
Present	20	39		33	30		30	28		31	26	
Child-Pugh score												
Class A	27	35	0.316	41	35	0.12	40	36	0.552	35	30	0.305
Class B	11	29		11	15		14	12		20	17	
Tumor number												
Single	25	37	0.306	38	36	0.632	35	36	0.145	40	32	0.411
Multiple	14	26		13	15		14	17		11	19	
Tumor size												
<5cm	28	39	0.205	42	37	0.78	40	32	O.558	44	30	0.553
≥5cm	14	31		10	13		18	12		15	13	
Tumor encapsulation												
Absent	10	25	0.078	21	20	0.065	19	21	0.894	22	18	0.059
Present	27	40		34	27		33	29		35	27	
Microvascular invasion												
Absent	23	38	0.052	40	28	0.001*	32	19	0.042*	30	21	0.762
Present	12	29		22	12		20	29		25	26	
Tumor differentiation												
I-II	21	40	0.061	30	25	0.049*	31	32	0.19	32	26	0.222
III-IV	11	30		19	28		21	18		20	24	
Intrahepatic metastasis												
Absent	10	31	0.001*	40	19	<0.001*	36	17	0.021*	32	15	0.012*
Present	18	43		32	11		29	20		35	20	
TNM stage												
I-II	22	46	0.059	36	28	0.12	32	34	0.106	30	35	0.114
III-IV	11	25		22	16		21	13		24	13	

* *P*<0.05

### Detection of natural Ct-HBx in hepatoma cells

We also examined the expression of full-length HBx and its truncated mutant in the eight hepatoma cell lines and the two immortalized normal liver cell lines using two pairs of PCR primers targeting different sequences of full-length HBx and HBxΔ31 (Figure 1C). The results showed that the full-length HBx transcript was detected only in HepG2.2.15 cells, whereas Ct-HBx mRNA was found only in MHCC-LM3 cells (Figure 1E), indicating that natural truncated HBx with the lost of 31aa at the C-terminal end (HBxΔ31) is expressed in MHCC-LM3 cells with high metastatic potential.

### Over-expression of Ct-HBx promotes invasion and metastasis of hepatoma cells *in vitro* and *in vivo*

**Figure 2:**
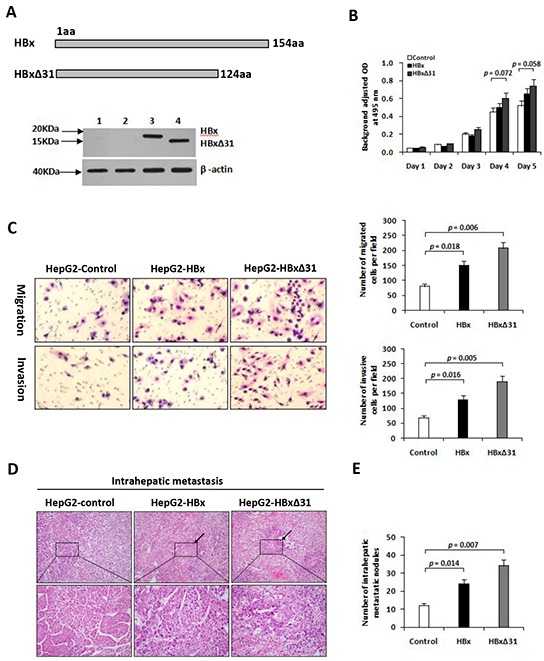
Over-expression of HBxΔ31 in HepG2 enhanced cell invasion and tumor metastasis **A.** Schematic diagram showing the different constructs of HBx used in this study. Western blot analysis of HBx expression in HepG2 cells stably transfected with HA-tagged HBx- or HBxΔ31-expressing plasmid and control vector pRc/CMV. β-actin was used as loading control. 1) Parental HepG2, 2) pRc/CMV, 3) Full-length HBx, 4) HBxΔ31. **B.** The growth rates of HBxΔ31- expressing cells was significantly increased compared to vector control, but had no effect on that of full-length HBx-expressing cells. **C.** The number of migrated or invaded cells was significantly increased in HBxΔ31-expressing cells, as compared to full-length HBx-expressing or vector control cells. Bars represent the numbers of migrated or invaded cells per field under 100 magnification. **D, E.** *In vivo* metastasis assay. (D) Representative images of livers from the different groups is shown. Black arrows indicate the intrahepatic metastatic tumors. (E) The number of metastatic nodules in the liver were counted and analyzed with Student's *t*-test (six mice per group). Results are derived from triplicate independent experiments (±SD).

We then established the stable cell lines, named HepG2-HBx and HepG2-HBxΔ31, after transfection of HA-tagged HBx or HBxΔ31 expressing vector. High expression of HBx gene was confirmed by Western blot analysis (Figure 2A). To determine the molecular mechanisms of the correlation between the presence of natural Ct-HBx and intrahepatic metastasis in HCCs, we analyzed the impact of full-length and HBxΔ31 on cell growth and cell migration and invasion ability in HCC cells. Transwell assays showed that both HepG2 cells expressing full-length and HBxΔ31 significantly enhanced cell migration and invasion ability compared to vector control cells. However, the number of migrated and invaded cells were more higher in HepG2 cells expressing HBxΔ31 than in HepG2 cells expressing full-length HBx (Figure 2C), suggesting that over-expression of HBxΔ31 promotes HCC cell migration and invasion. On the other hand, we also observed that ectopic expression of HBxΔ31 in MIHA hepatocytes lost the growth-suppressive effect of full-length HBx (Supplementary Figure S2B), but over-expression of HBxΔ31 did not change significantly the growth rates of HepG2 cells (Figure 2B). These results indicated that deletion of 31aa from C-terminus of HBx facilitates the cell migratory and invasive capacities but not affect on the cell growth at same situation in HCC. To excluding possible roles of cell lines, we investigated this effect of HBxΔ31 in Huh7 cells, and observed consistent with the effect of HBxΔ31 on the migration and invasion ability of HepG2 cells (Supplementary Figure S2B, S2C).

To further determine the role of HBxΔ31 in HCC metastasis *in vivo*, we established a animal model of HCC metastasis by implanting HCC cells expressing various forms of HBx into the livers of nude mice. Eight weeks after orthotopic implantation, the number of intrahepatic metastatic nodules was more over 2.0-fold increase in the mouse implanted with HepG2 cells expressing HBxΔ31 than in the mouse with HepG2 cells expressing full-length HBx (Figure 2D). Histological analysis further confirmed that over-expression of HBxΔ31 in HepG2 cells significantly enhanced the intrahepatic metastasis, as compared to both full-length HBx counterpart and vector control (51.2% vs. 30.8%, 51.2% vs. 18.2%, respectively) (Figure 2E). The results was consistently with over-expression of HBxΔ31 in Huh7 cells increased intrahepatic metastasis (Supplementary Figure S2D).

### Deregulation of metastasis-related proteins by Ct-HBx in hepatoma cells

**Figure 3:**
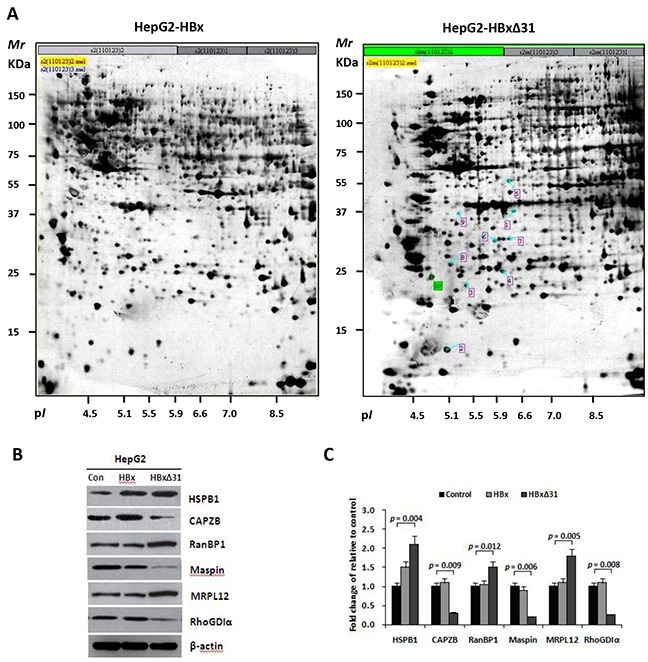
HBxΔ31 deregulates the metastasis-related proteins in HepG2 cells **A.** Representative silver-stained 2-DE profiles are shown from full-length HBx-expressing cells (HepG2-HBx, left) and HBxΔ31-expressing cells (HepG2-HBxΔ31, right). Proteins (400 μg) were focused over an IPG pH gradient of 4-7 and then separated by 10% acrylamide SDS-PAGE. Blue arrows indicate proteins that were deregulated proteins with at least 2-fold difference in HepG2-HBxΔ31 cells. Protein spots detected only in HepG2-HBxΔ31 cells are circled in red. **B.** Confirmation of the differentially-expressed proteins by Western blot analysis. Six proteins with divergent expression patterns between HepG2-HBx and HepG2-HBxΔ31 are shown. Comparing to full-length HBx, HBxΔ31 significantly up-regulated MRPL12, HSPB1 and RanBP1, whereas down-regulated Maspin, RhoGDIα and CAPZB in HepG2 cells. β-actin was used as an internal control. **C.** Fold changes of the differentially-expressed protein levels in HBx-expressing cell lines after normalization with β-actin are shown. Results are derived from triplicate independent experiments (±SD).

To investigate the machanistic basis of the distinctive effect of HBxΔ31 and full-length HBx on HCC cell invasion and metastasis, we performed proteomic analysis. Six proteins were displayed significantly differential expression (>2-fold change) in HepG2-HBxΔ31 compared to HepG2-HBx (Figure 3A), including up-regulated three proteins (MRPL12, RanBP1 and HSPB1), and down-regulated 3 proteins (Maspin, RhoGDIα and CAPZB) (Figure 3B and Table 2). Furthermore, Western blot analysis further validated the proteomic findings (Figure 3C). Intriguingly, some of HBxΔ31-repressed proteins, for example Maspin and RhoGDIα are reported to have anti-metastatic functions [23, 24], indicating a potential molecular mechanism that HBxΔ31 promotes HCC progression by reducing the expression of a subset of metastasis suppressors.

**Table 2:** Identification of the differentially expressed proteins in HepG2-HBx and HepG2-HBxΔ31 cells

Spot No.	Protein	Protein name	Mr(Da)	PI	Coverage	Score	Ratio**	Functions
	Accession*				(%)			
2	P52815	39S ribosomal protein L12, mitochondrial (MRPL12)	21,348	5.2	36	105	2.2	Cell growth
3	P47756	F-actin-capping protein subunit beta (CAPZB)	31,350	5.6	52	192	−3.1	Cell motility, migration and invasion
5	P04792	Heat shock protein beta-1 (HSPB1)	22,783	6.6	41	160	2.4	Cell growth, apoptosis and migration
6	P43487	Ran-specific GTPase-activating protein (RanBP1)	23,310	6.2	47	255	2.0	Cell growth
8	P52565	Rho GDP-dissociation inhibitor 1 (RhoGDIα)	23,207	5.3	38	156	−2.2	Cell motility, migration and invasion
9	P36952	Maspin (Serpin B5)	42,100	5.5	37	278	−2.6	Cell growth and metastasis

* Accession number of the UniProt database

** average ratio of HepG2-HBxΔ31 group/HepG2-HBx group.

### Maspin, RhoGDIα and CAPZB are transcriptionally repressed by Ct-HBx in HCC cells

**Figure 4:**
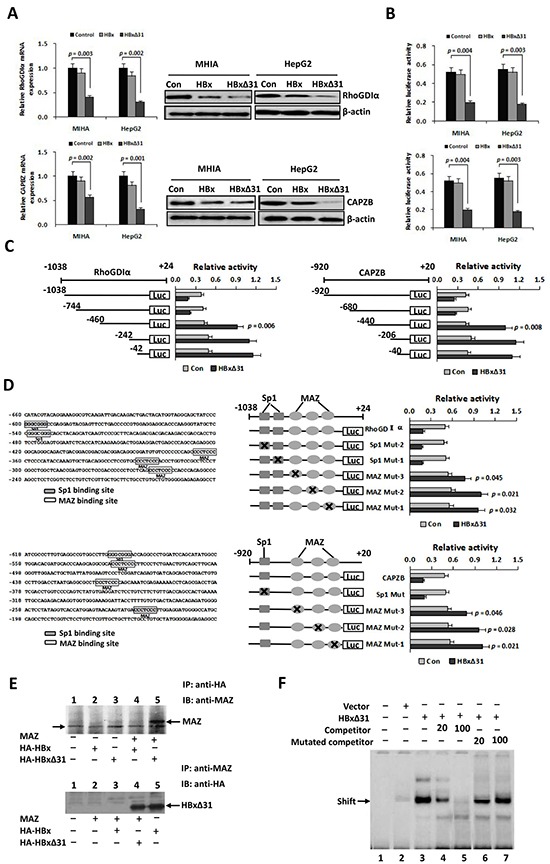
HBxΔ31 transcriptionally represses the RhoGDIα and CAPZB expressions through enhancing MAZ binding to the promoter **A.** Real-time PCR and Western blot analysis showing that HBxΔ31 repressed RhoGDIα and CAPZB expressions in MHIA and HepG2 cells. **B.** Dual luciferase report assay showing that HBxΔ31 repressed the RhoGDIα and CAPZB promoters. **C.** The sequence analysis demonstrated that the HBxΔ31 repressive element located between nt.-744 to −460 of the RhoGDIα promoter, while between nt. −680 to −440 of the CAPZB promoter. On the left panel, the schematic representation of the reporter gene constructs is shown; on the right panel, bars represent the relative promoter activity in each of the transfected cells. **D.** On the left panel, analysis of the cis-regulatory elements between nt. −744 to −460 in the RhoGDIα promoter revealed 2 Sp1 binding sites (GGGCGGG) and 3 MAZ binding sites (CCCTCCC), whereas between nt. −680 to −440 in the CAPZB promoter exhibited 1 Sp1 binding sites and 3 MAZ binding sites. On the right panel, MAZ sites in the RhoGDIα or CAPZB promoter was essential for HBxΔ31-induced RhoGDIα or CAPZB trans-suppression. Mutations in the Sp1 sites had no effect on the RhoGDIα and CAPZB promoter activities, whereas mutations in the MAZ sites significantly enhanced their promoter activities regulated by HBxΔ31. **E.** Coimmunoprecipitation assay showed a direct binding of HBxΔ31, but not full-length HBx, to MAZ in HCC cells. The arrow marks the location of a background band. **F.** EMSA assay confirmed enhancement of the DNA binding activity of MAZ to the RhoGDIα promoter by HBxΔ31.

To query whether HBxΔ31 could repress Maspin, RhoGDIα and CAPZB transcriptions in hepatoma cells, we performed real-time-PCR analysis. As a result, we found that over-expression of HBxΔ31 decreased the mRNA levels of Maspin, RhoGDIα and CAPZB in MIHA and HepG2 cells compared with full-length HBx and control vector (Figure 4A and Supplementary Figure S3A). Moreover, the protein levels of Maspin, RhoGDIα and CAPZB, as determined by Western blotting, were similarly reduced by HBxΔ31 compared with full-length HBx and control vector in MIHA and HepG2 cells (Figure 4A and Supplementary Figure S3B). In addition, luciferase reporter assay showed that promoter activities of Maspin, RhoGDIα and CAPZB were significantly decreased by HBxΔ31 in MIHA and HepG2 cells compared with full-length HBx counterpart and control vector (Figure 4B and Supplementary Figure S3C). Therefore, we conclude that Ct-HBx, rather than the full-length HBx counterpart, represses Maspin, RhoGDIα and CAPZB transcriptions by modulating their promoter activity.

### Lys-123 residue in HBxΔ31 is essential for the down-regulation of RhoGDIα and CAPZB expressions

To determine the region of HBxΔ31 involved in the suppression of RhoGDIα and CAPZB, we compared the suppression ability of two different Ct-HBx mutants, HBxΔ31 and hbxΔ31. HBxΔ31 is a HBx natural variant with 31aa truncated and accompanied with formation of 3 new amino acids at C-terminus, whereas hbxΔ31 is generated by a HBx artificial mutant with 31aa deleted at C-terminus from pRc/HBx (HBV adw2 subtype). As shown in Supplementary Figure S4A and S4B, two mutants generated completely diverse effects on the RhoGDIα and CAPZB expressions. HBxΔ31, but not hbxΔ31, was found to produce remarkable suppression effect on the RhoGDIα and CAPZB expressions. To further determine the critical amino acid residue in HBxΔ31 for this effect, we investigated whether the double substitution E121V and L123G was required for the distinct modulation of RhoGDIα and CAPZB expressions. Using three artificial HBxΔ31 variants with the substituted amino acid residues at 121st and/or 123th position in HBxΔ31, we found that only HBxΔ31 mutants changing codon 123 of the X protein from Lys to Glu (hbxΔ31L123G and hbxΔ31E121V/L123G) could effectively inhibit RhoGDIα and CAPZB expressions, up to the level obtained with HBxΔ31 (Supplementary Figure S4A), suggesting that Lys-123 is essential for the repressions of RhoGDIα and CAPZB.

### MAZ is critical for Ct-HBx-induced RhoGDIα and CAPZB trans-suppression

To define the roles of the *cis*-regulatory elements of the RhoGDIα and CAPZB promoters in response to HBxΔ31 regulation, we made a series of deleted variants of the RhoGDIα and CAPZB promoters and individually co-transfected with pCMV-HBxΔ31 or control vector into HepG2 cells (Figure 4C). The results of RhoGDIα deletion mutants showed HBxΔ31 still has repression on nt −1038 to −744, but lost its repression ability on nt −460, indicating that the HBxΔ31 repressive element localized between nt −744 and −460 of RhoGDIα promoter (Figure 4C). On the other hand, the results of CAPZB deletion mutants showed HBxΔ31 still has repression on nt −920 to −680, but lost its repression ability on nt −440, suggesting that the HBxΔ31 repressive element localized between nt −680 and −440 of CAPZB promoter (Figure 4C).

Using the Transcription Element Search Software program, analysis of the cis-regulatory elements between nt −744 to −460 of RhoGDIα promoter revealed 2 Sp1 binding sites (GGGCGGG) and 3 MAZ binding sites (CCCTCCC), while between nt −680 to −440 of CAPZB promoter had 1 Sp1 sites and 3 MAZ sites (Figure 4D). These HBxΔ31 repressive elements were changed by site-directed mutagenesis. The results showed that the mutation in Sp1 sites had no effect on the RhoGDIα or CAPZB promoter activity, whereas the mutation in MAZ sites significantly increased HBxΔ31-regulated RhoGDIα and CAPZB promoter activities (Figure 4D), indicating that the MAZ sites was required for their promoter inactivation regulated by HBxΔ31.

To determine whether HBxΔ31 enhanced transcriptional repression of MAZ, we examined interaction between MAZ and HBxΔ31 by coimmunoprecipitation experiment. Cells were co-transfected with MAZ and HA-tagged HBx or HBxΔ31 expressing plasmids, and then HBx were immunoprecipitated with anti-HA antibody. MAZ was detected in the immunoprecipitates by Western blot with anti-MAZ antibody. Reversely, HBx or HBxΔ31 was detected in immunoprecipitation with anti-MAZ antibody subjected to Western blot with anti-HA antibody. The results conformed that HBxΔ31, but not HBx, could directly associate with MAZ (Figure 4E).

To further determine the effect of HBxΔ31 on the recruitment of MAZ to the RhoGDIα and CAPZB promoters, we preformed Gel shift assay using an biotin-labeled oligonucleotide containing the MAZ binding sequence derived from the the RhoGDIα promoter (−252 to −262) was used as the probe. As shown in Figure 4F, enhanced binding to the wild-type oligonucleotide was observed in nuclear extracts from HBxΔ31-expressing HepG2 cells as compared with those from vector controls. We then analyzed the specificity of MAZ binding activity. The results showed that increased binding did not occur in the nuclear extract of HBxΔ31 incubated with the mutant oligonucleotides. The unlabeled wild-type oligonucleotides were able to compete with the labeled wild-type oligonucleotide, while the mutant oligonucleotides had no effect on the binding of the MAZ-HBxΔ31 complex to the labeled wild-type oligonucleotide, indicating HBxΔ31 represses RhoGDIα or CAPZB expression by enhancing MAZ binding to its consensus sequence.

### Maspin, RhoGDIα and CAPZB function to counteract HBxΔ31-induced invasion and metastasis of HCC cells

**Figure 5:**
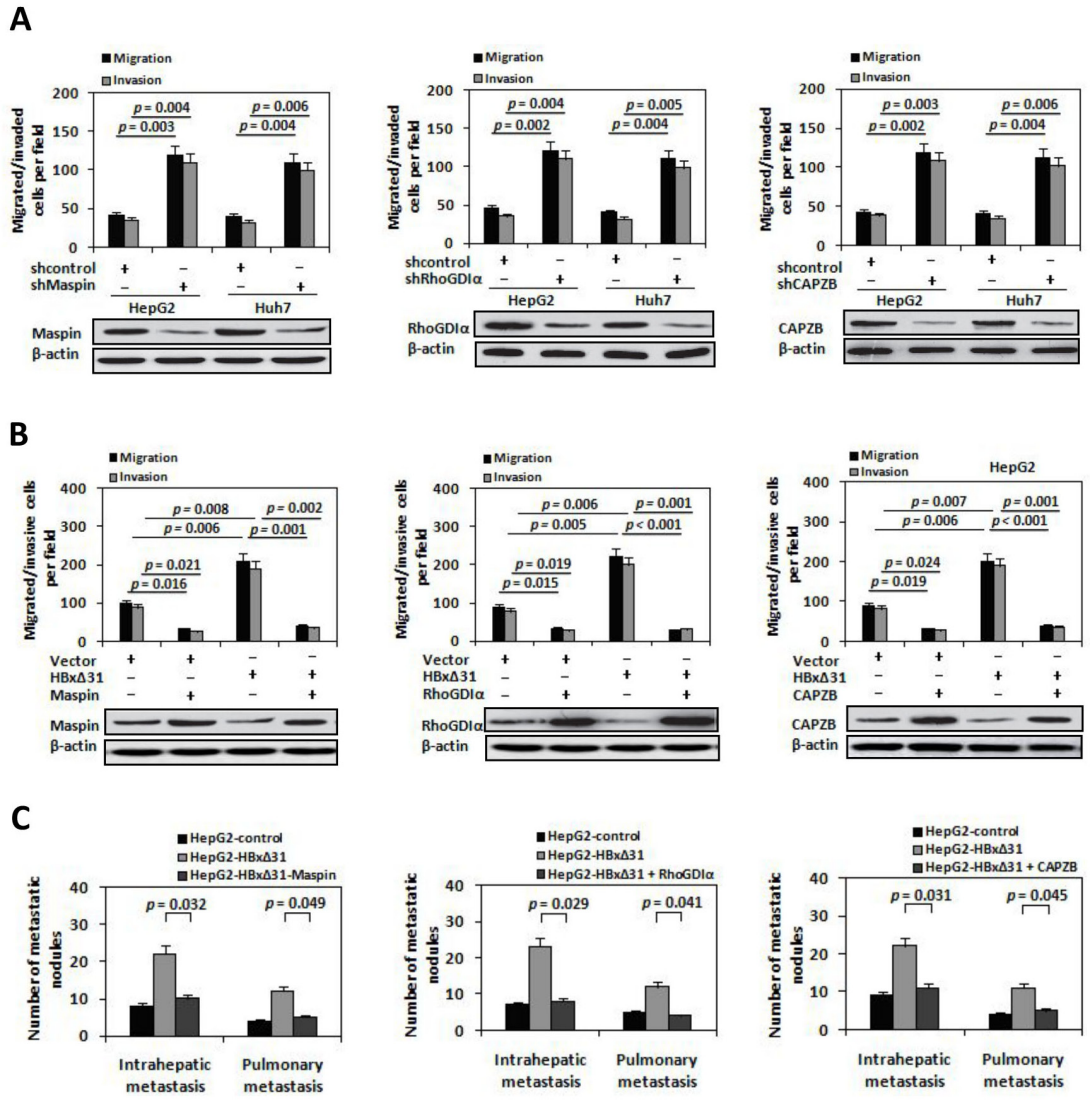
Maspin, RhoGDIα and CAPZB exert metastasis-inhibitory function in HBxΔ31-induced HCC cell invasion and metastasis **A.** The shRNA-mediated silencing of Maspin, RhoGDIα and CAPZB promoted HCC cell migration and invasion. HepG2 and Huh7 cells were infected with lentivirus-expressing Maspin shRNA (LV-shMaspin), RhoGDIα shRNA (LV-shRhoGDIα), CAPZB shRNA (LV-shCAPZB), or control shRNA (LV-shcontrol). On the upper panel, *In vitro* migration and invasion assays. On the lower panel, Western blot. **B.** Ectopic expressions of Maspin, RhoGDIα and CAPZB inhibited HBxΔ31-induced cell migration and invasion. HepG2 cells were co-transfected with lentivirus-expressing Maspin (LV-Maspin), RhoGDIα (LV-RhoGDIα), CAPZB (LV-CAPZB) or control (LV-control) and HBxΔ31-expressing plasmid or control vector. **C.** Suppression of Maspin, RhoGDIα and CAPZB expressions by HBxΔ31 increased the incidence of pulmonary and intrahepatic metastasis *in vivo*. The numbers of metastatic nodules in the lungs and liver in different groups of nude mice were counted and analyzed with Student's *t*-test (five mice per group). Results are derived from triplicate independent experiments (±SD).

To address the role of Maspin, RhoGDIα and CAPZB proteins in HCC metastasis, we abolished endogenous expression of Maspin, RhoGDIα or CAPZB using a short hairpin RNA (shRNA) in HepG2 cells, respectively. Inhibition of Maspin, RhoGDIα or CAPZB expression increased cell migratory and invasive activity (Figure 5A), which was similar to the phenotype induced by HBxΔ31 (Figure 2C and 5B). In contrast, HepG2 cells with ectopic expression of Maspin, RhoGDIα or CAPZB showed a 75% to 79% reduction of cell migration and invasion compared to vector control cells (Figure 5B).

To investigate whether Maspin, RhoGDIα and CAPZB exert the similar function in HBxΔ31-mediated HCC metastasis, we over-expressed HBxΔ31 with or without Maspin, RhoGDIα or CAPZB in HepG2 cells, and examined their metastasis both *in vitro* and *in vivo*. The results showed that HBxΔ31 enhanced both cell migration and invasion in HepG2 cells. Notably, HBxΔ31-induced cell migration and invasion were reduced upon co-expression of Maspin, RhoGDIα or CAPZB (Figure 5B). Concordantly, *in vivo* metastasis assay demonstrated pulmonary and intrahepatic metastasis nodules formed from HepG2-HBxΔ31 cells were significantly increased compared to that of control cells, whereas the numbers of metastasis-nodules formation of HepG2-HBxΔ31+Maspin, HepG2-HBxΔ31+RhoGDIα and HepG2-HBxΔ31+CAPZB cells were similar to that of control cells (Figure 5C). Consistent results were also observed in Huh7 cells. As shown in Supplementary Figure S5, down-regulation of Maspin, RhoGDIα and CAPZB expressions increased cell invasion and metastasis in Huh7 cells. Overall, these data demonstrate that Maspin, RhoGDIα and CAPZB function as the putative metastasis-suppressors in HCC and that are indeed the functional target for HBxΔ31.

### Ablation of Maspin, RhoGDIα and CAPZB correlate with C-terminal HBx truncation in HCC tissues

**Figure 6:**
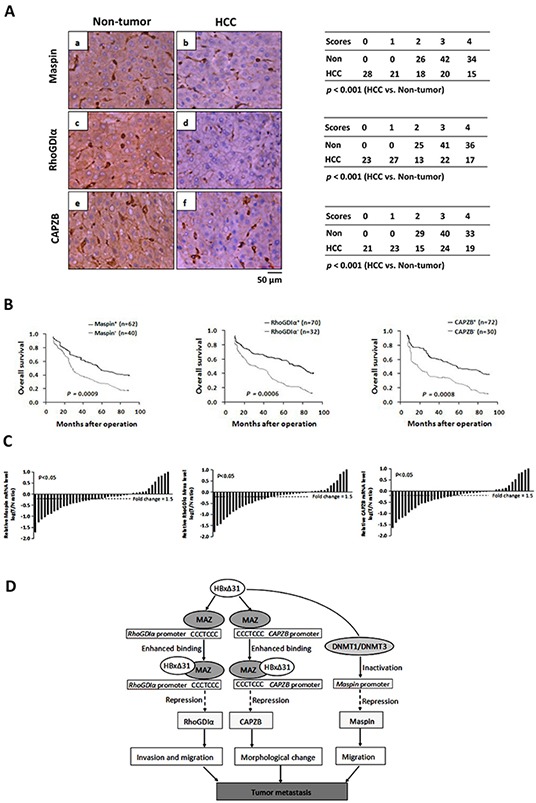
Down-regulation of Maspin, RhoGDIα and CAPZB is correlated with C-terminal truncated HBx in HCC samples **A.** Immunohistochemical staining of Maspin, RhoGDIα and CAPZB in HCC and adjacent non-tumorous tissue. (b) staining of Maspin in HCC-1, score 2; (a) non-tumorous tissue matched with HCC-1, score 4; (d) staining of RhoGDIα in HCC-2, score 2; (c) non-tumorous tissue matched with HCC-2, score 4; (f) staining of CAPZB in HCC-3, score 2; (e) non-tumorous tissue matched with HCC-3, score 4. The cases of each score were counted and are shown in the table. Statistical analysis was performed with Student's *t*-test. HCC, n = 102. For all three proteins, immunostaining was observed in the cytoplasm. Scale bar, 50μm. **B.** Kaplan-Meier analysis of the correlation between Maspin, RhoGDIα and CAPZB expressions and the overall survival of HBV-associated HCC patients. **C.** Comparison of Maspin, RhoGDIα and CAPZB mRNA expressions in 40 selected paired tumor (T) and non-tumorous (N) tissues which are only positive for HBxΔ31, using *PNN* as internal control. The bars (shown in log scale) illustrate the relative Maspin, RhoGDIα and CAPZB mRNA levels (T/N) in individual tissue pairs, of which negative and positive values respectively indicate down- and up-regulation of Maspin, RhoGDIα and CAPZB in HCCs. The differences of T and N groups are statistically significant (*p* < 0.05). **D.** A schematic diagram of the role of Maspin, RhoGDIα and CAPZB in the progression of HBV-associated HCC is shown.

To determine the clinical significance of Maspin, RhoGDIα and CAPZB down-regulation by HBxΔ31, we first examined the expression levels of Maspin, RhoGDIα and CAPZB in a cohort of 102 HBV-related HCC and their matching non-tumorous tissues using immunohistochemistry assay. The levels of Maspin, RhoGDIα and CAPZB were significantly lower in 60.8% (62 of 102), 68.6% (70 of 102) and 70.6% (72 of 102) of HCC tissues, respectively than the high basal levels of the matching non-tumorous tissues (Figure 6A) (*p* < 0.05). We then analyzed the relationship between the loss of Maspin, RhoGDIα and CAPZB expressions and clinical-pathological features. The results showed that Maspin, RhoGDIα and CAPZB down-regulation was correlated with multiple malignant characteristics, including microvascular invasion, malignant differentiation, and intrahepatic metastasis in HBV-associated HCC patients (Table 1). Kaplan-Meier analysis indicated that patients with low Maspin, RhoGDIα and CAPZB expressions had short overall survival than those with high Maspin, RhoGDIα and CAPZB expressions (Figure 6B), suggesting that reduced expressions of Maspin, RhoGDIα and CAPZB was implied in poor prognosis of HCC patients.

To further explore a link between HBxΔ31 expression and Maspin, RhoGDIα and CAPZB repressions in the same clinical specimens, we examined the mRNA levels of Maspin, RhoGDIα and CAPZB in 40 specific HCC specimens with HBxΔ31-positive by real-time PCR. The levels of Maspin, RhoGDIα and CAPZB were remarkably reduced in 70% (28 of 40), 72.5% (29 of 40) and 75% (30 of 40) of HCC tissues, respectively, as compared to their matching non-tumorous tissues (>1.5-fold change in 32 of 40 (80%), 31 of 40 (77.5%) and 33 of 40 (82.5%) of samples, respectively) (*p* < 0.05, Figure 6C), indicating that these metastasis-suppressors were at least partially suppressed by C-terminal truncation of HBx in HCC tissues.

## DISCUSSION

Previous studies have shown that HBx gene is frequent and spontaneous mutation in human HCC and leads to C-terminally truncated HBx (Ct-HBx) protein [6–9]. Nevertheless, such deletion of HBx is more often in tumors rather than their matching non-tumorous liver tissues. In this study, 70.6% (72 of 102) of HBV-associated HCC tissues contained Ct-HBx DNA and breakpoints between 120aa to 130aa was the major form of deletion (45.8% of the 72 cases) (Figure 1 and Supplementary Figure S1). Our result was consistent with that of a recent study showing that 79% of HCC from China had Ct-HBx transcript in tumor tissues [10]. These evidences indicate that C-terminally truncated mutation of HBx is frequent event in HCC. Interestingly, we identified two specific 8-nucleotides deleted mutations (codons 123-125 or 124-126) in HBx gene. All were accompanied with formation of a new stop codon and generated 31aa truncated at C-terminus, donated as HBxΔ31. It is noted that these truncation were different from the mutations previous reported within codons 128-134 of HBx sequence in HCC tissues [8, 9, 17]. Furthermore, HBxΔ31 was often over-expressed in HCC tissues, as compared with adjacent non-tumorous tissues and significantly correlated with its intrahepatic metastases (Figure 1 and Table 1), indicating that the presence of Ct-HBx has clinical significance.

We then attempted to illustrate the underlying mechanism of the correlation between the presence of HBxΔ31 and intrahepatic metastases in HCC by assessing cell invasion and metastasis. We showed that HBxΔ31 significantly enhanced HCC cell invasive and migratory activity compared with full-length HBx or control vector (Figure 2C and Supplementary Figure S2C). Moreover, the role of HBxΔ31 in cell invasion and migration is also evident in mouse hepatocytes, which increased intrahepatic metastatic nodules formation *in vivo* (Figure 2D and Supplementary Figure S2D), consistently demonstrating that HBxΔ31 play a significant role in promoting cell invasion and metastasis in HCC. In addition, we found that HBxΔ31 lost the growth-suppression effect induced of full-length HBx only in normal liver cell lines. However, the correlation between the over-expression of HBxΔ31 and cell growth did not been found in HCC cells (Figure 2B and Supplementary Figure S2B), suggesting that full-length HBx may be important in relation to anti-proliferation function, whereas its truncation with 31aa lost at C-terminal end mainly services as pro-metastasis effect in HCC cells. Further studies will be needed to confirm this notion.

To further explore the molecular basis of the divergent effect of full-length HBx and HBxΔ31 on HCC cell invasion and metastasis, we compared the proteomic profiles in HepG2 cells expressing full-length HBx and HBxΔ31. We identified that HBxΔ31 distinctively regulated 6 metastasis-related proteins, including three up-regulated and 3 down-regulated proteins, compared with full-length HBx (Figure 3 and Table 2). For example, HBxΔ31-up-regulated protein, soluble heat shock protein B1 (HSBP1), regulates VEGF-mediated angiogenesis through their direct interaction [18]. Regulation of HSBP1 on NF-κB pathway activation is involved in metastatic HCC cells apoptosis [19]. In contrast, HBxΔ31-down-regulated proteins, such as mammary serine protease inhibitor (Maspin) and Rho GDP dissociation inhibitor α (RhoGDIα), reveal anti-metastatic functions in various cancer types including HCC [20,21]. We further confirmed the metastasis-inhibitory effects of Maspin, RhoGDIα and CAPZB in hepatoma cells. These findings provide potential mechanism to insight into how Ct-HBx promotes cells invasion and metastasis in HCC.

During natural process of HBV infection, several mutations tend to be accumulated in the HBx gene coding sequence, which can impact its biological function and subsequent tumorigenic potential. Previous study demonstrated that some point mutations in HBx gene, such as the double substitution K130M and V131I, generated completely distinct impact on regulation of E-cadherin expression and cell migration [11]. In this study, we found that HBxΔ31, rather than full-length HBx, repressed Maspin, RhoGDIα and CAPZB expressions at transcription initial steps by inducing their promoter inactivation. Further analysis showed that HBxΔ31 coding region at 120-125aa residues produces a double substitution E121V to L123G mutations in contrast to full-length HBx gene (Figure 4 and Supplementary Figure S3). Interestingly, only the HBxΔ31 variants containing Lys-123 instead of Glu-123 (L123G) effectively reduced RhoGDIα and CAPZB expressions (Supplementary Figure S4A-S4B). Lys-123 has been shown to localize in the trans-activation domain of HBx [4]. Therefore, we speculate that Lys-123-containing HBxΔ31 may more effectively regulate transcription suppressor MAZ expression, resulting in RhoGDIα and CAPZB promoter inactivation. Further investigation should be warranted.

HBx is essential for trans-activation of HBV and host cell gene. Despite having no binding to DNA directly, HBx can deregulate cellular gene expression by interacting with transcription regulators in the nucleus [2–4, 22]. Previous reports showed that full-length HBx can transcriptionally suppress a subset of important tumor-related genes through enhancing the promoters binding to transcription suppressors like E2F1, SMAD4, YY1 and MAZ [23–25]. In this report, deletion and mutation analysis of the RhoGDIα and CAPZB promoters revealed that the MAZ binding sites is critical for HBxΔ31-mediated RhoGDIα and CAPZB suppression (Figure 4C and 4D). However, HBxΔ31 did not affect binding of Sp1 to its consensus sequence in the RhoGDIα or CAPZB promoters, while the RhoGDIα or CAPZB promoter activities were increased when the MAZ binding sites were mutated in HBxΔ31-expressing cells, suggesting that MAZ is a suppressor of the RhoGDIα or CAPZB promoter. Coimmunoprecipitation experiment showed that HBxΔ31, but not full-length HBx, physical associated with MAZ (Figure 4E). EMSA assays further confirmed that HBxΔ31 enhanced the DNA binding activity of MAZ to the RhoGDIα and CAPZB promoters (Figure 4F). Thus, HBxΔ31 repressed RhoGDIα and CAPZB expressions by enhancing the binding of MAZ to the RhoGDIα and CAPZB promoters through its physical association with MAZ. The results was consistent with previous report, which HBx with point mutation at C-terminus acts as a transcriptional co-repressor on telomerase promoter by enhance MAZ binding to the promoter [24], suggesting that C-terminus of HBx may play a critical role in trans-suppression of the specific cancer-related gene. In addition, we identified that amino acids 104 to 124 domain of HBxΔ31 is required for the binding to MAZ to the promoter region of RhoGDIα and CAPZB (Supplementary Figure S4C-S4E). These findings indicated that Ct-HBx-dependent ablation of RhoGDIα and CAPZB through MAZ may be one of major reasons underlying the decreased expressions of RhoGDIα and CAPZB in hepatoma cells.

Interestingly, besides HBxΔ31 interacting with MAZ resulting in RhoGDIα down-regulation, over-expressed miR-151 can repress RhoGDIα expression and facilitate tumor cell migration and spreading in HCC [28]. It has been reported that CpG site promoter methylation led to Maspin down-regulation in tumors [29, 30]. Our unpublished data also revealed that HBxΔ31 represses Maspin expression by inducing promoter hypermethylation through activation of DNA methyltransferase 1 and in turn promotes cell invasion and metastasis in HCC. Thus, in addition to Ct-HBx, other mechanism might be involved in suppression of Maspin, RhoGDIα or CAPZB in HCC cells. Further studies are warranted.

The loss of expression or function of metastasis-suppressor genes is a critical inducer for triggering a series of metastatic events initiate [32]. According to previous studies, Maspin, RhoGDIα and CAPZB are often deregulated in many malignancies and significantly correlated with metastasis, recurrence and prognosis [31, 37–39]. Functional studies further delineated that Maspin, RhoGDIα and CAPZB play important roles during tumor metastasis by influencing cell morphology, adhesion junctions and migration ability of tumor cells [32, 33, 36]. However, the functional link between Maspin, RhoGDIα along with CAPZB and HBV-related HCC metastasis has not been explored so far. In this report, we found that Maspin, RhoGDIα and CAPZB were reduced expressions in two HCC cell lines and human HCC tissues (Figure 2 and Figure 6). Subsequent functional analyses further demonstrated that Maspin, RhoGDIα and CAPZB act to impede the invasion and metastasis of HCC cells *in vitro* and *in vivo* (Figure 5 and Supplementary Figure S5). Moreover, we demonstrated that Maspin, RhoGDIα and CAPZB expressions were significantly lower in HCC tissues with C-terminal truncation of HBx than the matching non-tumorous liver tissues (Figure 6) and associated with metastatic characteristics and poor prognosis in HCC patients (Table 1 and Figure 6). These findings establish a metastasis-suppressive role of Maspin, RhoGDIα and CAPZB in HCC, and indicate that down-regulation of Maspin, RhoGDIα and CAPZB is vital for Ct-HBx-mediated progression of hepatocarcinogensis.

Maspin is a member of the serine protease inhibitor (serpin) superfamily and displays tumor-suppressing activity by controlling cell migration, proliferation, apoptosis, and adhesion, whose down-regulation was significantly correlated with poor prognosis of patients in several types of tumors [30, 32]. Recent report showed that down-regulation of Maspin by miRNA-7/21/107 confers HBx-mediated aggressiveness in HCC [37]. In our present study, we verified that HBxΔ31-dependent suppression of Maspin by inducing inactivation of its promoter resulted in the enhancement of invasion and metastasis of HCC cells.

Another important metastasis-suppressor transcriptionally repressed by Ct-HBx is RhoGDIα, a regulator of Rho activity, controls on Rho family GTPases, including Rac1, Cdc42 and Rho GTPases activation. RhoGDIα/Rac1 signal pathway plays an essential role in regulating cell morphology, adhesion, and motility. The aberrant expression of RhoGDIα was related to tumor metastasis and poor clinical outcomes in aggressive cancers [21, 33, 34]. In this study, we found that ectopic expression of RhoGDIα reduced HBxΔ31-induced cell invasion and metastasis. Moreover, RhoGDIα was a direct transcriptional and functional target of HBxΔ31. These data strongly suggested that RhoGDIα contribute to HBxΔ31-induced HCC progression.

Cell migration often requires profound changes in cellular architecture, with protrusion of different structures such as pseudopodia, filopodia, or lamellipodia [35, 36]. F-actin capping protein (CP), a heterodimer composed of α and β subunits, inhibits actin filaments elongation by binding to barbed ends of growing actin filaments, which is a critical step for creating actin architecture of lamellipodia and consequential cell protrusion and motility [37–39]. Reports on the role of CP in cancer are rare thus far. A recent study showed that CPα1 subunit (CAPZA1) down-regulation in gastric cancer was associated with poor prognosis and with increased cancer cell migration and invasion [40]. In this report, we show for the first time that CPβ subunit (CAPZB) was repressed by Ct-HBx in HCC. Functional assays revealed that the forced expression of CAPZB resulted in a significant reduction for HBxΔ31-expressing HepG2 cell migration and invasion. These findings demonstrated that CAPZB functions to counteract HBxΔ31-induced cell invasion and metastasis. Taken together, Ct-HBx may alter cell migratory and invasive ability and subsequently promote HCC metastasis through repressing metastasis suppressors and interfering their corresponding gene-network.

In conclusion, our data show that C-terminal truncation of HBx, particularly with breakpoint at 124 amino acids (HBxΔ31), can significantly promote HCC cell invasion and metastasis *in vitro* and *in vivo*. Meanwhile, HBxΔ31 exerts this function via specifically suppressing a set of metastasis-suppressors (Maspin, RhoGDIα and CAPZB) expressions, in part, by enhancing their promoter binding to transcription suppressor MAZ through its physical association with MAZ. This newly identified HBxΔ31/Maspin, RhoGDIα or CAPZB module provides a new insight into an understanding of the progression of hepatocarcinogenesis, especially invasion and metastasis, and may facilitate the development of potential therapeutics against HBV-associated HCC.

## MATERIALS AND METHODS

### Patients and samples

One hundred two pairs of human HCCs and their corresponding non-tumorous liver tissues from chronic HBV-infection patients with liver resection for HCC between 2005 and 2012 at the First Affiliated Hospital of Guangzhou Medical University (Guangzhou, China), were randomly selected for study. These 102 patients had positive serum hepatitis B surface antigen (HBsAg) status. Patients' ages ranged from 39 to 72 years; 76 were male and 26 female. All specimens were snap-frozen in liquid nitrogen and kept at −80°C. Frozen sections were cut from non-tumorous liver and tumor blocks separately and stained for histological examination to ensure a homogenous cell population of tissues. The accompanied intrahepatic metastases were observed through both naked eyes and microscope. Follow-up data were summarized at the end of December 2012 with a median follow-up of 44 months (range 6-88 months). Human tissue samples were collected with written consent of the patients and prior approval from Clinical Research Ethics Committee of Guangzhou Medical University. The clinical characteristics of the patients are listed in Table 1.

### Cell lines

HCC cell lines including HepG2, HepG2.2.15, Hep3B, Huh7, SMMC7721, MHCC97L, MHCC97H and MHCCLM3 and two immortalized normal liver cell lines LO2 and MIHA were purchased from the American Type Culture Collection (ATCC). All cells were maintained in high-glucose cultured in Dulbecco's modified Eagle's medium (DMEM, Gibco) supplemented with 10% fetal bovine serum (HyClone).

### Plasmids

Full-length HBx DNA (adw2 subtype; NCBI-X02763) was amplified from HBx/pcDNA3.1^+^ plasmid and subcloned into HA/pCMV6-AN vectors. HBx truncation mutant with 31aa delated at C-terminus (denoted HBxΔ31) was made and subcloned into HA/pCMV6-AN vectors. Wild-type RhoGDIα (−1038 to +24), CAPZB (−920 to +20) and Maspin (−467 to +50) promoters were amplified from healthy human liver DNA, respectively. The mutants with mutations at the putative Sp1 or MAZ transcription factor binding sites on the RhoGDIα or CAPZB promoters were subcloned into pGL3-Basic vector. MAZ-expressing plasmid was made by introducing the corresponding complementary DNAs (cDNAs) into pRc/CMV vector. Lentiviral vectors encoding short hairpin RNAs (shRNAs) were generated with the use of PLKO.1-TRC (Addegene) and were designated as LV-shRhoGDIα, LV-shCAPZB, LV-shMaspin and LV-shcontrol, respectively. Lentiviral vectors encoding human RhoGDIα, CAPZB and Maspin gene were constructed in FUW-teto (Addegene), designated as LV-RhoGDIα, LV-CAPZB and LV-Maspin. The empty vector was used as negative control, designated as LV-control. All primers are shown in Supplementary Table S1.

### Establishment of HBx-expressing cells

HepG2 and Huh7 cells were transfected with HA-tagged HBx and HBxΔ31 expressing plasmids or control vector using Lipofectamine 2000 (Invitrogen), according to manufacture's protocol. HBx-expressing cells were selected with G418 at 700 μg/mL for 14 days. The individual clones were confirmed to express HBx by western blot analysis using anti-HA antibody.

### Two-dimensional electrophoresis

Prior to two-dimensional electrophoresis, the protein samples were purified using a 2D Clean-Up kit (GE healthcare) according to the manufacturer's instructions. Differentially expressed proteins were identified using two-dimensional gel electrophoresis and mass spectrometry. Two-dimensional gel electrophoresis was performed using immobiline strips (p*I* range, 3-10; GE Healthcare) with proteins being separated according to charge and subsequently molecular weight. The gels were then silver stained in order to visualize proteins and the differentially expressed spots were identified by MALDIPMF mass spectrometry.

### Western blot analysis

Cells were lyzed in sodium dodecyl sulfate (SDS) containing buffer and equal amounts of protein were separated in SDS/polyacrylamide gel electrophoresis gel for western blotting analysis. Immunodetection was performed using anti-HA, anti-MAZ and anti-β-actin (Santa Cruz), **anti**-HSPB1 and anti-CAPZB (LSBio), anti-Maspin and anti-RhoGDIα (Abcam), anti-MRPL12 and anti-RanBP1(Cell Signaling Technology).

### Luciferase reporter assay

HepG2 cells were transfected with different combinations of plasmids using FuGENE 6 reagent (Roche), according to the manufacturer's protocol. Plasmids used included HA/pCMV6-AN vector containing various forms of HBx, RhoGDIα-WT/pGL3-Basic, RhoGDIα-MAZ-Mut/pGL3-Basic and CAPZB-WT/pGL3-Basic, CAPZB-MAZ-Mut/pGL3-Basic reporter constructs, and an internal control (pRL-SV40). The total amount of expression vectors was equalized with the empty vector. Twenty four hours after transfection, luciferase and Renilla luciferase activities were measured by the Dual Luciferase Reporter assay system (Promega), according to the manufacturer's protocol. Transfection efficiency was normalized with the Renilla luciferase activity. Experiments were done thrice independently.

### Coimmunoprecipitation assay

Cells were cotransfected with pCMV-MAZ and either pCMV-HA/HBx or pCMV-HA/HBxΔ31. Forty-eight hours after transfection, cells were lysed in TBS containing 0.5% NP-40. The cell debris was removed by a brief centrifugation in a microcentrifuge. The supernatant was then immunoprecipitated with anti-HA antibody. The immunocomplex precipitated with Pansorbin was then analyzed by Western blotting with anti-MAZ primary antibody. Alternatively, the cell lysates were first immunoprecipitated with anti-MAZ antibody, followed by Western blotting with the anti-HA primary antibody.

### Electrophoretic mobility shift assay (EMSA)

The preparation of the HepG2 cells nuclear extracts and the gel shift assay were described previously [15]. The biotin end-labeled double-strand oligonucleotide probe used for the gel shift contained the sequence 5′-AGCCCTCCCCA-3′ (−252 to −262 in RhoGDIα promoter) or a mutant oligonucleotide 5′-AGCCCTATACA-3′ covering the putative MAZ-binding sites at the promoter region was employed for standard PCR measurement in the EMSA assay. EMSA was performed using the LightShift® Chemiluminescent EMSA Kit (Pierce) according to the manufacturer's protocol.

### *In vitro* migration and invasion assay

For the migration assays, 5×10^4^ cells were placed into the top chamber of each insert. For the invasion assay, 1×10^5^ cells were added to the upper chamber of each insert coated with 150 μg of Matrigel. After several hours of incubation at 37°C, cells that had migrated or invaded were fixed and stained in dye solution containing 0.1% crystal violet and 20% methanol. The cells that had migrated or invaded were counted and imaged using an inverted microscope (Olympus).

### *In vivo* metastasis assay

BALB/C nude mice (6-8 weeks) were housed under standard conditions and cared for according to the institutional guidelines for animal care. All animal experiments were approved by the Guangdong Province Medical Experimental Animal Care Commission. For *in vivo* metastasis assays, 4×10^6^ cells in phosphate-buffered saline to a volume equivalent to 8% of the total body weight of each animal were injected subcutaneously into the flanks of nude mice. After 4 weeks, the subcutaneous tumors were removed and diced into 1 mm^3^ cubes, which were then implanted in the left lobes of the livers of the nude mice (six per group). After 8 weeks, mice were sacrificed, and their livers and lungs were dissected and prepared for standard histological examination.

### Statistical analysis

For clinic-pathological correlation analysis, Fisher's exact test was used for analysis of categorical data. For *in vitro* cell migration and invasion assay and reporter assay, the Student *t* test was used for continuous data. Two-sided *P* values less than 0.05 were considered as statistically significant.

## SUPPLEMENTARY FIGURES AND TABLES


