# Supplementary material for: Carboxyl-terminal truncated HBx contributes to invasion and metastasis via deregulating metastasis suppressors in hepatocellular carcinoma
Source: Oncotarget. 2016 Jul 4;7(34):55110–27. doi: 10.18632/oncotarget.10399 (PMC5342405; doi:10.18632/oncotarget.10399)
Supplement: Supplementary file 1 [file oncotarget-07-55110-s001.pdf]

Carboxyl-terminal truncated HBx contributes to invasion and metastasis via deregulating metastasis suppressors in hepatocellular carcinoma

SUPPLEMENTARY MATERIALS AND METHODS

DNA and RNA isolation, reverse transcription polymerase chain reaction

Total DNA was extracted from frozen HCC and the matching non-tumor liver tissues by a Quick-Gene DNA Tissue Kits (Qiagen). Total RNA was extracted from samples using Trizol reagent (Invitrogen), according to manufacture's protocol. For polymerase chain reaction (PCR) amplification of HBx, sets of PCR primers (1F: 5′-ATGGCTGCTAGGCTGTGCT-3′, 285R: 5′-CTTATGAAGACCTTGGGCAC-3′ and 465R: 5′-TTA GGCAGAGGTGAAAAAGTTGC-3′) were used for full-length and Ct-HBx, respectively (Figure. 1A). In addition, to detect the presence of truncation at 130, 140, and 150aa of Ct-HBx, respectively, sets of PCR primers (1F: 5′-ATGGCTGCTAGGCTGTGCT-3′, 390R: 5′-ATCTAATCTCCTCCCCCAAC-3′, 420R: 5′-CAATTTATGCCTACAGCCTCCTAC-3′ and 450R: 5′-TTAGTTGCATGGTGCTGGTGCGCAG-3′) were used (Supplementary Figure. 1A).

A set of PCR primers was used to amplify RhoGDIα, CAPZB and Maspin cDNAs, respectively, β-actin used as a reference for the amount of cDNA added in the PCR reactions. Quantitative and semi-quantitative reverse transcription-PCR (RT-PCR) were performed as previously described [16]. The primers used are shown in Supplementary Table S1. Amplified PCR products were subjected to DNA sequencing.

Real-time PCR

Total RNA was extracted using Tirzol reagent, (Invitrogen), and reverse transcription was performed using a PrimeScript RT-PCR Kit (Takara)" according to the manufacturer's instructions. For the real-time PCR analysis, aliquots of double-stranded cDNA were amplified using a SYBR Green PCR Kit (Applied Biosystems). The cycling parameters were 95°C for 15 seconds, 55°C for 15 seconds, and 72°C for 15 seconds for 45 cycles. A melting curve analysis was then performed. Ct was measured during the exponential amplification phase, and the amplification plots were analyzed using SDS 1.9.1 software (Applied Biosystems). For the cell lines, the relative expression level (defined as fold change) of the target gene was determined by the following equation: $2^{-\Delta\Delta Ct}$ ($\Delta Ct = \Delta Ct^{target} - \Delta Ct^{GAPDH}$; $\Delta\Delta Ct = \Delta Ct^{expressing\ vector} - \Delta Ct^{control\ vector}$). The expression level was normalized to the fold change detected in the corresponding control cells, which was defined as 1.0. For clinical tissue samples, the fold change of the target gene was determined by the following equation: $2^{-\Delta\Delta Ct}$ ($\Delta\Delta Ct= \Delta Ct^{tumor} - \Delta Ct^{nontumor}$), and it was normalized to the average fold change in the normal liver tissues, which was defined as 1.0. All reactions were performed in duplicate. Primer sequences are listed in Supplementary Table S1.

Cell proliferation assay

Cell viability was assessed by a colorimetric method using Cell Titer 96®AQueous One Solution Cell Proliferation Assay (Promega, Madison, WI) for 5 consecutive days. Two thousand cells were seeded to 96-well plate in 6 replicates. The plate was light-protected and incubated at 37°C for 1 hour. The absorbance of the colorimetric products formed was measured at 495nm by a spectrophotometer.

Immunohistochemistry

HBV-related HCC samples and the corresponding adjacent liver tissues (n=102) were used for the measurement of RhoGDIα, CAPZB and Maspin protein expression by immunohistochemistry assay. Immunohistochemical staining was performed with the Dako Envision Plus System (Dako, Carpinteria, CA) according to the manufacturer's instructions. The final score of each sample (negative or positive) was assessed by summarization of the results of the intensity and extent of staining. Intensity of staining was scored as 0 (negative), 1 (weak), or 2 (strong). The extent of staining was based on the percentage of positive tumor cells: 0 (negative), 1 (1%-25%), 2 (26%-50%), 3 (51%-75%), and 4 (76%-100%). Therefore, each case was finally considered negative if the final score was 0 to 1 (-) or 2 to 3 (±) and positive if the final score was 4 to 5 (+) or 6 to 7 (++), respectively.

SUPPLEMENTARY FIGURES AND TABLE

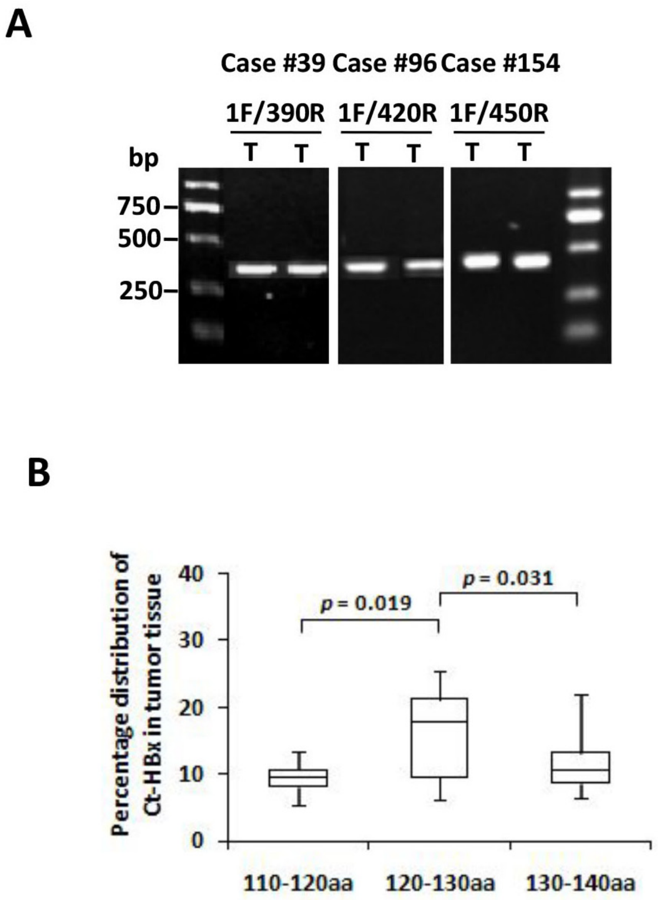

Supplementary Figure S1: Common HBx DNA breakpoints in our HCC samples were located between 120aa to 130aa of C-terminal end of HBx. A. Representative results showing the presence of Ct-HBx with breakpoints between 120aa to 130aa in the HCC cDNA samples. Case 39 and 96 had C-terminal truncation at breakpoints between 120aa and 130aa, whereas Case 154 showed presence of C-terminal truncation between 130aa and 140aa. **B.** Pie chart showing the percentage distribution of C-terminal truncated HBx in HCC tumor samples.

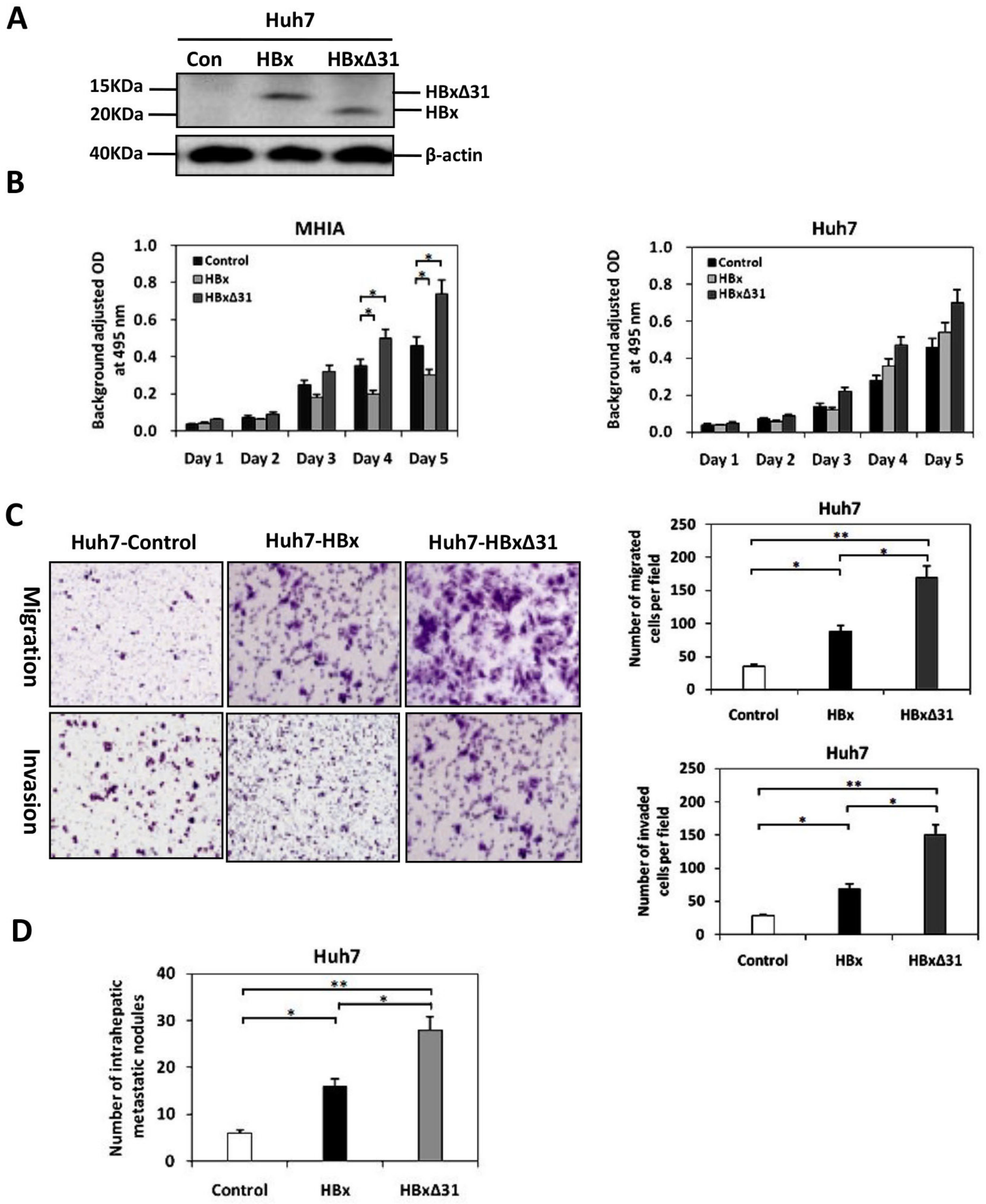

Supplementary Figure S2: Expression and functions of HBxΔ31 in Huh7 cells. A. Western blot analysis of full-length HBx and HBxΔ31 expressions in Huh7 cells transfected with the control vector, HA-tagged full-length HBx or HBxΔ31 expressing plasmid. **B.** Growth curves of stable MHIA and Huh7 cells expressing full-length HBx or HBxΔ31. Although ectopic expression of HBxΔ31 in the normal liver cell line, MHIA, lost the growth-suppressive effect of the full-length HBx, mild expression of full-length or HBxΔ31did not show a significant alteration of cell-growth rates in the HBx-expressing Huh7 cells. **C.** Transwell assays were performed in the stable Huh7 cells expressing full-length HBx or HBxΔ31.The numbers of both migrated cells and invaded cells were significantly increased in HBxΔ31-expressing cells as compared with full-length HBx-expressing cells. **D.** *In vivo* metastatic assay. Huh7 cells expressing full-length HBx or HBxΔ31were transplanted into the livers of nude mice. The number of metastatic nodules in the liver was significantly increased in group transplanting with HBxΔ31-expressing cells as compared with in group transplanting with full-length HBx-expressing cells. *P<0.05, **P<0.01.

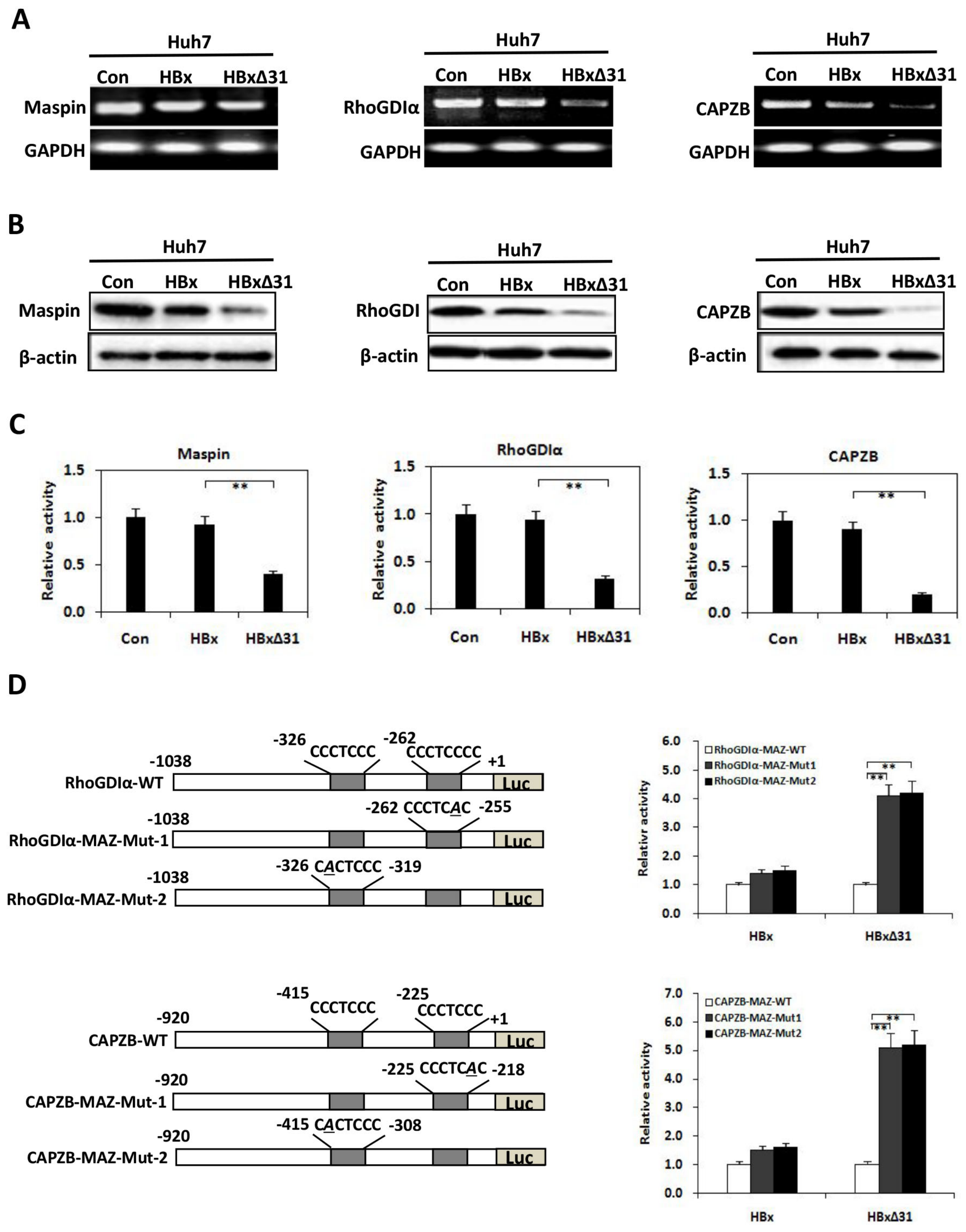

Supplementary Figure S3: HBxΔ31 represses Maspin, RhoGDIα and CAPZB expressions by inducing promoter inactivation. A. Semi-quantitative RT-PCR measurement of Maspin, RhoGDIα and CAPZB mRNA transcripts in full-length HBx- and HBxΔ31-expressing Huh7 cells. **B.** Western blotting confirmed similar proteins expression levels of Maspin, RhoGDIα and CAPZB in corresponding full-length HBx- and HBxΔ31-expressing Huh7 cells. **C.** Dual luciferase reporter assay showing that HBxΔ31 reduced the Maspin, RhoGDIα and CAPZB promoter activities in Huh7 cells. **D.** HBxΔ31 repressed the WT RhoGDIα and CAPZB promoters, but not the RhoGDIα and CAPZB promoter, having one of mutations of the MAZ-binding sites in Huh7 cells. Bars represent mean values of relative promoter activity from three independent experiments.

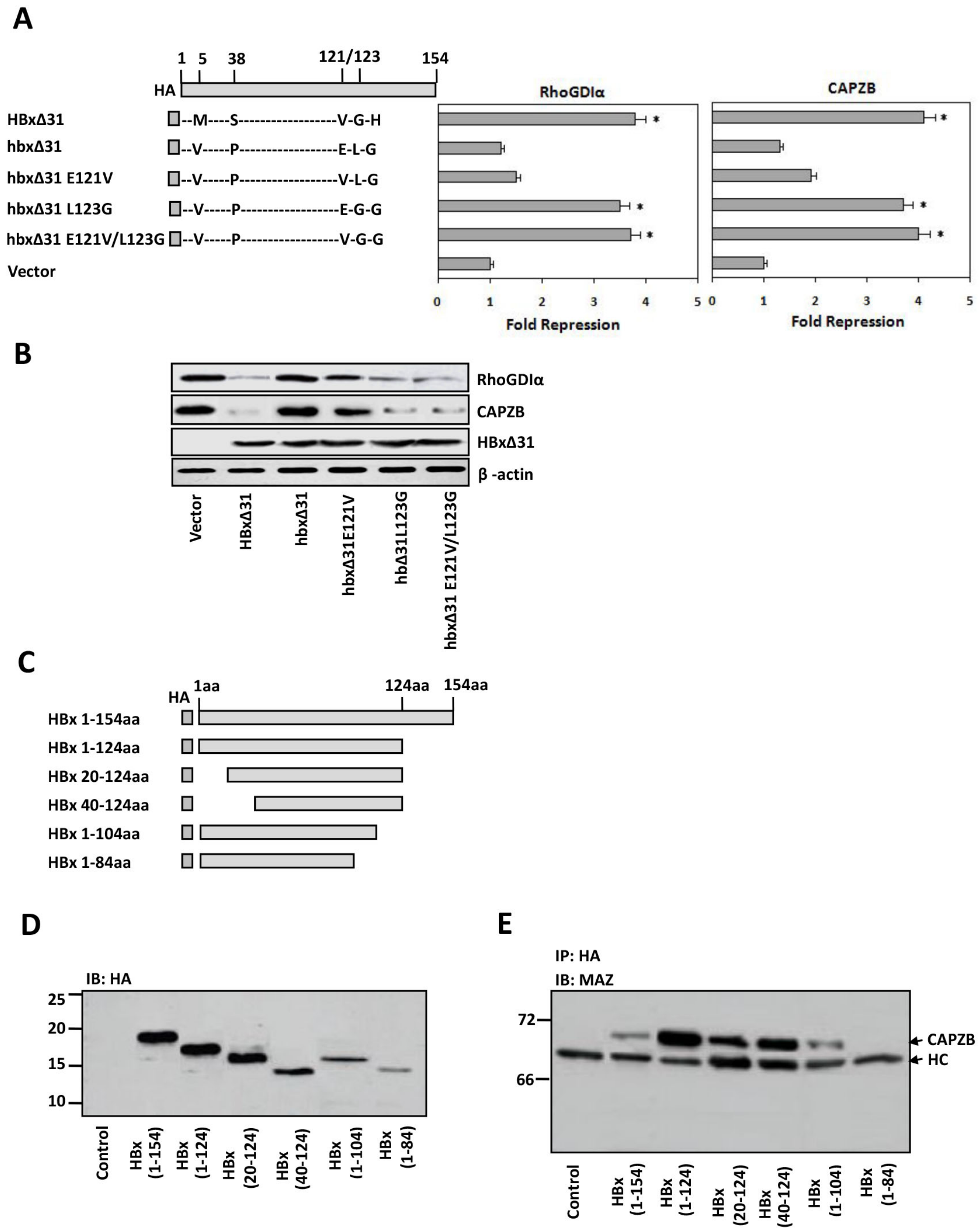

Supplementary Figure S4: Identification of the HBxΔ31 domain critical for the RhoGDIα and CAPZB repression. A. HBx natural variant with 31aa truncated at C-terminus (HBxΔ31), HBx artificial mutant with 31aa deleted at C-terminus from wild type HBV adw2 subtype (hbxΔ31) and hbxΔ31 derivative mutants are schematically represented. The amino acid residues that are different between HBxΔ31 and hbxΔ31 are indicated. Each Ct-HBx-expressing construct was cotransfected with the RhoGDIα or CAPZB promoter report construction and luciferase assay was performed. Repression fold was calculated by dividing the luciferase activity of Ct-HBx-expressing cells by the basal activity of the control. **B.** Western blot analysis of RhoGDIα and CAPZB in HepG2 cells transiently transfected with Ct-HBx construct was performed. β-actin was used as an internal control. **Elucidation of the domains of HBxΔ31 required for interaction with MAZ.** HBxΔ31 deletions were generated and tested for interaction with MAZ by immunoprecipitation. **C.** Schematic representation of the HA-tagged HBxΔ31 deletions generated. aa, amino acids. **D.** HA-tagged HBxΔ31 deletions were transfected into HepG2 cells, and the lysates derived from these cells were separated by SDS-PAGE and transferred to PVDF membranes for immunoblotting (IB) with anti-HA antibody. **E.** HBxΔ31 deletions were transfected into HepG2 cells and immunoprecipitated (IP) with anti-HA antibodies. Immunoprecipitates were separated by SDS-PAGE and transferred to PVDF membranes for immunoblotting with anti-MAZ antibody. Interactions between HBxΔ31 and MAZ require amino acids 104 to 124. HC, IgG heavy chains.

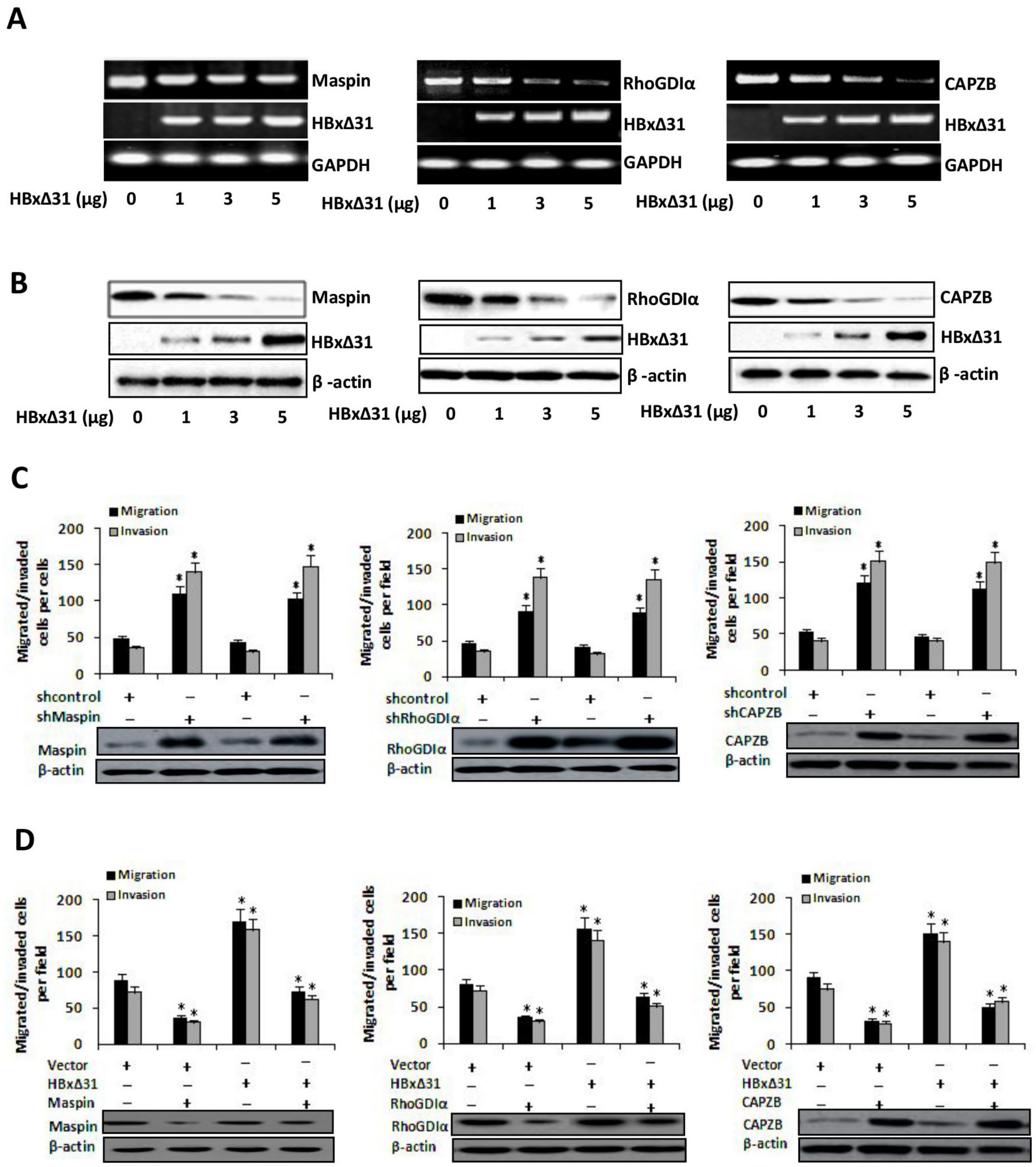

Supplementary Figure S5: HBxΔ31 facilitates Huh7 cell migration and invasion by reducing the Maspin, RhoGDIα and CAPZB expressions. **A.** Representative results on semi-quantitative RT-PCR measurement of Maspin, RhoGDIα and CAPZB transcripts in HBxΔ31-expressing Huh7 cells. **B.** Protein levels of HBxΔ31 and Maspin, RhoGDIα and CAPZB were confirmed by Western blotting. **C.** Reduced expression of Maspin, RhoGDIα and CAPZB promoted Huh7 cell migration and invasion. **D.** Ectopic expressions of Maspin, RhoGDIα and CAPZB inhibited HBxΔ31-induced Huh7 cell migration and invasion.

Supplementary Table S1: Primers sequences for PCR and constructs

Gene Name	Forward primer	Reverse primer
1F/285R(F1/R1)	5′-ATGGCTGCTAGGCTGTGCT-3′	5′-CTTATGAAGACCTTGGGCAC-3′
1F/390R(F1/R2)	5′-ATGGCTGCTAGGCTGTGCT-3′	5′-ATCTAATCTCCTCCCCCAAC-3′
1F/420R(F1/R3)	5′-ATGGCTGCTAGGCTGTGCT-3′	5′-CAATTTATGCCTACAGCCTCCTAC-3′
1F/450R(F1/R4)	5′-ATGGCTGCTAGGCTGTGCT-3′	5′-TTAGTTGCATGGTGCTGGTGCGCAG-3′
1F/465R(F1/R5)	5′-ATGGCTGCTAGGCTGTGCT-3′	5′-TTAGGCAGAGGTGAAAAAGTTGC-3′
1F/372R	5′-ATGGCTGCTAGGCTGTGCT-3′	5′-CCCCCAACTCCTCCCAGTGTTTA-3′
1F/465R	5′-ATGGCTGCTAGGCTGTGCT-3′	5′-TTAGGCAGAGGTGAAAAAGTTGC-3′
Maspin qPCR	5′-GGATGTAAACAAACTTAGTTCC-3′	5′-ATGCAGGTCAAGGAAGAGATC-3′
RhoGDIα qPCR	5′- GTTTGTGCTGAAGGAGGGTGT-3′	5′-TCGTCTGTGAAGCGGGACT-3′
CAPZB qPCR	5′-CGCCTTCTTTGCAGCCTTTGGG-3′	5′-AGTATGGAGGCTCAAGTAAG-3′
β-actin qPCR	5′- TTGTTACAGGAAGTCCCTTGCC-3′	5′-ATGCTATCACCTCCCCTGTGTG-3′
Maspin ORF	5′-CGGGATCCATGTGAAAAGGAGCCACTGG-3′	5′-ATAATATTTAGGCTCAACAGCAATG-3′
RhoGDIα ORF	5′-CGGGATCCATGGCTGAGCAG GAGCCCAC-3′	5′-CCGCTCGAGCTCAGTCCTT CCAGTCCTTC-3′
CAPZB ORF	5′-AGCTAAGCTTCCACCATG GCCGACTTCGA-3′	5′-AATTGAATTCTTAAGCAT TCTGCATTTCT-3′
MAZ ORF	5′-TCCCCGCTGCGGCCGAGGCC-3′	5′-CGGGAGAAGCTCTTGCCACAG-3′
β-actin ORF	5′-GTCACTTCAGCTCCTTTCCT-3′	5′-ATCTTGCGAAAGGCGGAACT-3′
